# Undiagnosed pediatric type 2 diabetes mellitus: the giant hidden iceberg

**DOI:** 10.1007/s12519-025-00917-3

**Published:** 2025-06-03

**Authors:** Jia-Qi Pu, Jun-Fen Fu

**Affiliations:** Department of Endocrinology, Children’s Hospital, Zhejiang University School of Medicine, National Clinical Research Center for Child Health, Hangzhou, 310051 China

Owing to the increasing prevalence and severity of obesity in childhood and adolescence, the incidence and prevalence of prediabetes and type 2 diabetes mellitus (T2DM) in children and adolescents are increasing worldwide. However, the incidence of clinically diagnosed pediatric T2DM remains low, representing the “tip of the iceberg”. A long asymptomatic period of diabetes, a lack of awareness of risk factors, and a shortage of mass screening instruments result in the underdiagnosis of T2DM, especially in children. Compared with adult T2DM and type 1 diabetes mellitus (T1DM) patients, pediatric T2DM patients have a more aggressive phenotype and a greater risk of morbidity, increasing the clinical and economic burden. Here, we discuss the prevalence of pediatric T2DM and prediabetes, with a focus on undiagnosed T2DM. We also discuss potential screening tools that may aid in pediatric T2DM identification.

## The rise of pediatric T2DM

In recent decades, the prevalence of diabetes in children has significantly increased globally. Notably, the rates of prediabetes and T2DM among adolescents have risen dramatically, paralleling the rapid increase in childhood obesity [1–4]. In 2021 alone, about 41,600 new cases of T2DM were diagnosed in children and adolescents worldwide, indicating the growing burden of this condition [5]. China has experienced one of the most prominent increases in diabetes prevalence worldwide. The prevalence of T2DM in Chinese children increased 2.5-fold from 1995 to 2010 (4.1–10.0 per 100,000 persons) [6]. Similarly, a more than 5.5-fold increase in the prevalence of T2DM was observed in Korea among children aged 5–19 years from 2002 to 2016 [7]. In the United States, the increase in the incidence of T2DM among people aged 10–19 years was 7.1% from 2002 to 2012 [4], whereas an annual increase exceeding 10% in both indigenous and nonindigenous children from 1990 to 2012 was documented in Western Australia [8]. The multinational Better control in Pediatric and Adolescent diabeteS: Working to crEate CEnTers of Reference (SWEET registry), encompassing data from five regions between 2012 and 2021, further highlighted a 9% rise in the relative rate of newly diagnosed T2DM per two-year period [9]. Equally alarming is the shifting paradigm in the epidemiology of diabetes among youth. Accumulating evidence indicates that the incidence of T2DM is greater than that of T1DM [4]. In the United States, the SEARCH for Diabetes in Youth (SEARCH) study revealed that T2DM accounts for about half or more of newly diagnosed diabetes cases across different ethnic groups among adolescents aged 10–19 years [10]. This trend is mirrored elsewhere, and the proportion of new-onset T2DM is reported to be as high as 66% in Indigenous Australians [11] and 68.6% in China [6].

T2DM has emerged as a silent epidemic that is often undetected and underdiagnosed because its insidious progression is characterized by prolonged insulin resistance and β-cell dysfunction. The International Diabetes Federation (IDF) Diabetes Atlas reported that almost one in two (44.7%) adults aged 20–79 years with diabetes were unaware of their diabetes status [12]. This pattern is observed globally. An analysis from the UK Office for National Statistics between 2013 and 2019 estimated that about 30% of the adults living with T2DM remained undiagnosed. Younger adults were more likely to be undiagnosed (50% of those aged 16–44 years and 27% of those aged 75 years and over) [13]. Similarly, a report from Sweden revealed that nearly half of the individuals who developed T2DM during a 10-year period were undiagnosed [14]. In the USA, the National Diabetes Statistics Report revealed that 28% of adults with diabetes were unaware of their condition [13]. The situation is also concerning in China, where a population-based study revealed that only 36.5% of adults with diabetes were aware of their diagnosis [15].

T1DM has long been regarded as the predominant form of pediatric diabetes, overshadowing T2DM in both clinical focus and public awareness. However, emerging evidence has challenged this paradigm. Our recent nationwide Prevalence and Risk of Obesity and Diabetes in Youth (PRODY) study, which included 193,801 Chinese children and adolescents aged 3–18 years, indicated that self-reported diagnosed cases were predominantly T1DM (70.83%), whereas T2DM accounted for 29.17%. However, population-based screening data revealed that the prevalence of undiagnosed T2DM was almost tenfold higher than that of self-reported T2DM, revealing a previously unrecognized large fraction of pediatric diabetes cases [16]. This phenomenon is not confined to China. In the United States, data from the National Health and Nutrition Examination Survey (NHANES) 1999–2010 revealed that T2DM accounts for about half of cases of adolescent diabetes in the USA, with 34% remaining undiagnosed [2]. A multicenter study conducted in central Europe (Germany, Austria, and Switzerland) estimated that about 6000 children suffer from T2DM, while only 1/6 of them were officially registered [17], indicating a significant underdiagnosis of the disease.

Collectively, these findings underscore a critical shift in the epidemiology of pediatric diabetes, and the high prevalence of undiagnosed pediatric T2DM represents a hidden iceberg, requiring an urgent need for enhanced screening.

## Complications and mortality of pediatric T2DM

The increasing incidence and prevalence of pediatric T2DM and the high proportion of undiagnosed cases place a tremendous burden on the healthcare system at present and in the future. Moreover, T2DM that develops in childhood or adolescence has been reported to be more aggressive than adult-onset T2DM, with a greater risk of long-term complications and comorbidities, as well as premature mortality and poorer overall health and quality of life, than childhood T1DM [18, 19].

The Treatment Options for Type 2 Diabetes in Adolescents and Youth (TODAY) study demonstrated that the majority of youth experienced complications after a 15-year follow-up period [20]. At baseline, 8% of the youth participants in TODAY had diabetic kidney disease (DKD); 19.2% had hypertension; and 20.8% had dyslipidemia. The proportions increased to 54.8% for DKD, 67.5% for hypertension, and 51.6% for dyslipidemia after 15 years, revealing the rapid and early accumulation of complications in pediatric diabetes patients [20]. The SEARCH study also revealed that youth-onset T2DM patients have a substantially greater risk of both microvascular and macrovascular complications than do T1DM patients of similar age and diabetes duration [21]. DKD is the most common and earliest complication in adolescent T2DM patients, with a significantly greater risk of progression than in adult T2DM patients and young T1DM patients with similar disease durations [22]. Renal disease is also a critical risk factor for cardiovascular disease (CVD), the leading cause of death among adults with T2DM. The morbidity and mortality associated with CVD in youth with T2DM have been intensely investigated. Evidence suggests that compared with their adult counterparts, adolescents diagnosed with T2DM face twice the risk of macrovascular complications. Data from Australia also demonstrated that young-onset T2DM is associated with more diabetes complications, especially macrovascular disease [23]. Age at diagnosis of T2DM is prognostically important for survival. Studies indicate that survival is more than a decade shorter if T2DM is diagnosed in adolescence [24]. Furthermore, patients diagnosed with T2DM between 15 and 30 years of age have a significantly higher mortality rate than do those with T1DM, despite a shorter disease duration [23].

Building on the alarming clinical phenotype of pediatric T2DM, its underlying pathophysiology further underscores the urgency of addressing this growing crisis. Mechanically, individuals diagnosed with T2DM at a young age have more rapid deterioration of β-cell function. The TODAY study revealed that the average decline in β-cell function in youth with T2DM was nearly 2–3-fold greater than that in adults (20%–35% vs. 7%–11%) [25]. Adolescents have severe insulin insensitivity but show a greater loss of glucose-stimulated insulin secretion, collectively causing earlier β-cell failure and poor glycemic control than those observed in adult-onset T2DM [26]. The more rapid deterioration of β-cell function likely explains the aggressive phenotype of pediatric T2DM, characterized by rapid loss of glycemic control and higher HbA1c levels. These distinct features highlight the need to prevent diabetes, and if possible, to reverse the development of pediatric T2DM. Evidence from adults has revealed that early interventions can delay or prevent progression to T2DM and its complications, although evidence in youth is limited [27, 28]. The Restoring Insulin Secretion (RISE) Pediatric Medication Study reported that long-term medications help prevent worsening of oral glucose tolerance test (OGTT) parameters in youth aged 10–19 years with impaired glucose tolerance (IGT) or newly diagnosed T2DM.

Overall, pediatric T2DM presents a uniquely severe and aggressive phenotype, distinct from adult-onset T2DM or childhood T1DM, and imposes a substantial tremendous burden on the public healthcare system. Thus, there is an urgent need to identify prediabetes and early diabetes in children and adolescents.

## Screening of pediatric T2DM

In view of the increased prevalence and severity of pediatric T2DM, the identification of high-risk youth for early diagnosis and intervention has become a critical public health priority, as younger age at diagnosis is potentially protective [19]. Considering the substantial population potentially affected by pediatric T2DM, routine screening for T2DM in primary care is urgently needed. However, the development and validation of practical, evidence-based screening strategies remain an unmet clinical need.

Current guidelines for T2DM issued by leading organizations all recommend a risk factor-based strategy for screening for pediatric T2DM [29–31]. However, these guidelines do not provide a unified screening method. For the identification of high-risk populations, identifying risk factors strongly associated with pediatric T2DM is challenging. Non-modifiable markers such as family history, ethnic background and early-life determinants such as maternal gestational diabetes are relatively straightforward to ascertain. However, it is difficult to assess modifiable risk factors, such as disordered eating, unhealthy lifestyles (increased sedentary time, low physical activity and excess screen time) and comorbidities [dyslipidemia, metabolic syndrome, polycystic ovary syndrome (PCOS), non-alcoholic fatty liver disease (NAFLD), etc.], which play important roles in both T2DM screening and prevention. Thus, there is an urgent need for the development of effective, child-specific assessment tools.

In the tests for diagnosing diabetes, fasting plasma glucose (FPG), OGTT, and HbA1c continue to be pivotal. HbA1c has multiple advantages, including elimination of the need for fasting, high stability, standardized measurement, and long-term association with the future development of diabetes. However, its sensitivity in children has been reported to be lower than that in adults (as low as 0%–5% in children vs. 23%–27% in adults) [32]. A screening in US children and adolescents revealed that the sensitivity and specificity for detecting any hyperglycemia of HbA1c and FPG were both low (HbA1c: sensitivity = 55.5%, specificity = 76.3%; FPG: sensitivity = 35.8%, specificity = 77.1%) [33]. The OGTT, regarded as the gold standard for diagnosing diabetes, presents its own set of challenges. Its lengthy testing duration and the requirement for multiple blood draws (4–5 times) render it impractical for large-scale screening initiatives.

This limitation underscores the need for more efficient and scalable screening tools, particularly in pediatric populations where early detection is critical. Our recent research introduced a simplified OGTT (s-OGTT) as a promising alternative for population-based screening. By measuring capillary blood glucose during fasting and two hours post-administration, the s-OGTT not only detects impaired fasting glucose (IFG) but also identifies IGT. This approach has proven effective in identifying a substantial number of undiagnosed pediatric T2DM and prediabetes patients, demonstrating its feasibility as a screening method. The screening is recommended for overweight (BMI ≥ 85th percentile) or obese (BMI ≥ 95th percentile) children and adolescents (aged > 10 years or at onset of puberty) with one or more additional risk factors, including family history of T2DM (including gestational diabetes), high-risk ethnicity, signs of insulin resistance or conditions associated with metabolic syndrome. Annual screening should be performed if the results are within range. The frequency of screening may need to be shortened to half a year if prediabetes is present or if the risk profile is worsening. Population-based screening is usually expensive, and its benefits and harms are difficult to assess. Thus, other potential diagnostic markers, such as urinary myo-inositol, salivary glucose and fluorescent probes detecting dipeptide peptidase IV (DPP-IV), are potential indicators for the early detection of diabetes [34–36]. Moreover, two-step screening, which involves the use of risk assessment tools to identify high-risk populations and then a diagnostic test to identify individuals with diabetes, may be a viable alternative. As the global burden of diabetes continues to rise, further research is imperative to refine the timing and methods of screening.

The incidence of T2DM in children and adolescents is significantly increasing worldwide, even exceeding that of T1DM in some populations. Remarkably, a high proportion of children with T2DM are living with unawareness. The combination of early complications, longer disease duration, poor glycemic control, and faster reduction in β-cell reserve leads to a more severe and aggressive disease phenotype and increased cumulative disease burden among adolescents with T2DM. Thus, it is particularly important to identify children with undiagnosed T2DM as well as those at the highest risk of developing T2DM. More research should be conducted into the development of effective strategies for early screening and intervention.

## Data Availability

Not required.
